# Growth challenges and recovery in 1247 children with congenital diaphragmatic hernia: a 10-year follow-up

**DOI:** 10.1007/s00431-025-06479-w

**Published:** 2025-11-07

**Authors:** Alexander J. Jordan, Christoph Mohr, Richard Martel, Katrin Zahn, Thomas Schaible, Rüdiger Adam, Michael Boettcher, Julia Elrod

**Affiliations:** 1 Department of Pediatrics, Pediatric Gastroenterology, University Medical Center Mannheim, Heidelberg University, Theodor-Kutzer-Ufer 1-3, Mannheim, 68167 Germany; 2 Department of Pediatric Surgery, University Medical Center Mannheim, Heidelberg University, Theodor-Kutzer-Ufer 1-3, Mannheim, 68167 Germany; 3 Department of Neonatology and Pediatric Intensive Care, University Medical Center Mannheim, Heidelberg University, Theodor-Kutzer-Ufer 1-3, Mannheim, 68167 Germany

**Keywords:** CDH, Growth, Weight, Length, BMI, Head circumference

## Abstract

Growth abnormalities in children with congenital diaphragmatic hernia (CDH) are a significant concern for long-term developmental outcomes. To explore growth in this population, the evolution of weight, height, body mass index (BMI), and head circumference as vital indicators of growth and developmental progress in CDH survivors were investigated. We analyzed 1247 CDH patients treated at our center from 2000 to 2022. Growth trends regarding weight, height, BMI, and head circumference of surviving patients without major comorbidities were compared with standard pediatric growth milestones for term and preterm children. Factors associated with growth disparities were assessed. A total of 835 CDH survivors without major comorbidities were identified who exhibited notably slower weight gain, which stabilized at a reduced level beyond the age of 2 years. By 6 years of age, height normalized in term-born children, while head circumference normalized at 1 year of age. BMI remained low throughout childhood. Defect size and gestational age were important independent predictors of growth restriction, whereas ECMO therapy was not an independent risk factor for low weight or small stature. Preterm children tended to exhibit lower growth percentiles and delayed catch-up.

*Conclusion* This study reveals that while initial growth is typically delayed in CDH survivors, a significant number achieve catch-up in childhood. The results lay the groundwork for personalized nutritional and therapeutic strategies to enhance growth outcomes in children with CDH.

**Table:** 

**What is Known:**	
• *Children with congenital diaphragmatic hernia frequently experience early growth failure and undernutrition.*	
• *Existing studies include ≤ 200 patients and tend to provide only short-term follow-up data.*	
**What is New:**	
• *A cohort of 1247 CDH patients was treated at our center; in survivors without major comorbidities, growth was evaluated repeatedly up to 14 years.*	
• *Height and head circumference normalize, whereas weight and BMI remain lower; large defect size and lower gestational age, but not ECMO, predict poor growth.*

**Supplementary Information:**

The online version contains supplementary material available at 10.1007/s00431-025-06479-w.

## Introduction

Congenital diaphragmatic hernia (CDH) is a condition arising from an abnormal development of the diaphragm, resulting in a defect of varying size. The defect allows abdominal organs to herniate into the thoracic cavity. This condition is typically characterized by lung hypoplasia, altered pulmonary vasculature, and cardiac abnormalities such as left ventricular hypoplasia. About 50–60% of CDH cases occur as isolated defects, while the rest are associated with genetic anomalies, syndromes, or other concomitant malformations [1–3]. Children with a prenatal diagnosis of CDH are usually intubated immediately after birth [4–6]. If conventional treatment is insufficient, extracorporeal membrane oxygenation (ECMO) may be necessary [7]. Following the initial treatment, various complications such as chronic lung disease with increased metabolic demands, gastroesophageal reflux disease (GERD), and feeding difficulties persist [2]. Children are at risk of growth impairment and malnutrition, which could adversely affect future development [8]. 

Several small studies comprising fewer than 60 CDH patients each have reported failure to thrive in up to 69% of patients at the age of 1 year [9–11]. In the last decade, studies with slightly larger cohorts were published: A multicenter retrospective observational study reported growth impairment in 19.5% of children with CDH at an age of 18 months [12]. In one of the largest prospective studies to date, with 172 CDH patients monitored up to the age of 12 years, weight-for-height remained below average until the age of 5 years in non-ECMO and 8 years in ECMO patients, respectively. Subsequently, both groups experienced catch-up growth, however, patients remained smaller up to the age of 12 years compared to the norm irrespective of ECMO use [13].

Previous studies demonstrate that failure to thrive is common in CDH patients; however, they tend to be heterogeneous regarding specific outcomes, extent of catch-up growth, and the role of ECMO as an independent risk factor. Most studies are based on substantially smaller cohorts and often lack long-term follow-up. BMI and head circumference (HC) are rarely analyzed, representing additional gaps. This study addresses these gaps by evaluating comprehensive growth parameters in a large, longitudinal cohort and identifying predictors of growth impairment.

The present prospective study comprises an analysis of growth among 1247 CDH patients treated at a national referral center between 2000 and 2022, with data collected up to 18 years of age, though not all participants completed the entire follow-up**.** The study’s objective is to provide the basis for the refinement of treatment and nutrition guidelines for children with CDH.

## Methods

### Study population and ethics

A prospective cohort study was conducted at the Children’s University Medical Center Mannheim, Heidelberg University, between January 2000 and December 2022, on patients diagnosed with CDH. Data acquisition encompassed all inpatient admissions, outpatient clinical encounters, and appointments within the structured lifelong follow-up program. The dataset incorporated an array of variables, including patient demographics, CDH-specific clinical parameters, and details pertinent to therapeutic interventions. Ethical approval was granted by the Institutional Review Board of Heidelberg University (ID: 2022–626 and ID: 2018-592N-MA). Informed consent was obtained from all participants or their legal guardians in adherence to the Declaration of Helsinki.

### Standard of care for CDH

Patients were either born at the University Medical Center Mannheim, transferred after birth, or joined the follow-up program at a later time point. Starting in 2007, standardized postnatal management was performed according to the CDH Euro Consortium Consensus, updated in 2015 [5, 14].

### Follow-up-program and assessment of growth data

All participants were systematically enrolled in a comprehensive, longitudinal follow-up protocol. The protocol encompassed an interdisciplinary evaluation at predetermined intervals: 6 and 12 months, and 2, 4, 6, 10, 14, and 18 years of age, as described previously [15]. Anthropometric measurements including weight and height were documented at each visit, while HC was recorded only until the age of 24 months. Due to limited data availability, no formal statistical analysis was performed beyond the 10-year follow-up point; however, individual data points up to 14 years are shown for descriptive purposes.

### Growth percentiles

WHO Child Growth Standards were used for weight, height, BMI, and HC of children aged 0–5 years [16]. Conversely, for children aged ≥ 5, growth standards of the Centers for Disease Control and Prevention (CDC) were applied [17, 18]. Fenton preterm infant growth charts were applied for the calculation of *z*-scores of birth weight, height, and HC for all children with a gestational week < 40 + 0 to enable plotting measurements at their exact corresponding gestational week and day [19, 20].

### Statistics

Statistical analysis was performed using Python (version 3.11, 2024) and the statsmodels library (version 0.14.2, 2024), as well as SPSS (SPSS 29, 2022). Graphs were created using the Python libraries matplotlib (version 3.10, 2025) and seaborn (version 0.13, 2025). *Z*-scores were calculated based on the LMS formula [21]. For the determination of mean *z*-scores at the time of standardized follow-up, only measurements within a specific tolerance range were included. These were 6 months ± 2 months; 12 months ± 3 months; 2 years ± 3 months; 4 years ± 6 months; 6 years ± 6 months, and 10 years ± 12 months, respectively.

For infants born before 40 + 0 weeks of gestation, chronological age was corrected for the expected date of birth. Calculations encompassed term and preterm infants without significant comorbidities. Relevant comorbidities were defined as any diseases with an increased likelihood of affecting growth, as well as multisystem diseases and syndromes. Minor comorbidities not expected to affect growth, like hemodynamically non-relevant cardiac septal defect, were not excluded.

For plausibility testing of the growth data, *z*-scores below − 5 or above + 2 were manually reviewed in the clinic’s patient information system. Implausible values were corrected for any potential input errors. The *Z*-test was applied to each age cohort to assess differences from the expected means regarding weight, height, HC, and BMI. Accounting for repeated measurements over time, a linear mixed model (LMM) was used to evaluate changes in weight and height between the 6-month and 6-year time points. In the model, the *z*-score was used as the dependent variable, time of measurement as a fixed effect, and patients as a random effect. The model was estimated using the Restricted Maximum Likelihood (REML) method.

Descriptive statistics are presented as means with standard deviations. Two-tailed t-tests were used to identify differences between groups. Multiple linear regression was utilized to identify factors associated with suboptimal growth patterns. Propensity score matching (PSM) was used to control for confounding factors in order to evaluate whether ECMO influences weight gain and height. Here, 1:1 matching of ECMO and non-ECMO patients was performed, resulting in two groups with no significant differences regarding the baseline characteristics, gender, gestational age, defect size, and defect laterality, respectively. Missing data were not replaced or imputed. Analyses at each follow-up time point were based on the subset of patients with available data within the defined time windows. The number of patients per time point is reported in the corresponding tables. The significance threshold was set at *p* < 0.05.

## Results

### Study population

A total of 1247 patients were included in the analysis over a period of 23 years (see Supplementary Material 1). Of these, 286 (22.9%) died during the observation period. Of the 286 patients who died, 245 (85.7%) did so within the first 30 days of life, and 31 (10.8%) between 1 and 12 months. In six cases, the exact date of death was unknown.

Of the 961 surviving patients, 868 (90.3%) had no relevant comorbidity, whereas 93 (9.7%) had concurrent medical problems such as clinically suspected or genetically proven syndromes, major cardiac anomalies, major malformations, or other diseases affecting growth. To characterize growth in children with CDH, the study focused on patients without additional comorbidities. Of the 868 surviving patients without a relevant comorbidity, 666 were born at term and 169 were preterm infants; for 33 patients, this information was missing. This resulted in a cohort of 835 patients with detailed information for analysis. Note that the rate of children with large defects and consecutive need for patch repair of the diaphragm is rather high in the cohort treated at our hospital, see also Table 1. Follow-up completeness decreased over time, with fewer patients reaching later time points (see Tables 2 and 3 and Online Resources 7 and 9). A comparison between patients with and without available follow-up data at 10 years is shown in Online Resource 2, revealing a difference only in defect size B and C, but not in any other baseline characteristic.

**Table 1:** Patient characteristics

	Term patients without relevant comorbidities and surviving only	Preterm patients without relevant comorbidities and surviving only	***P***-value
Total	666	169	
Gestational age [week]	38.54 ± 1.06	34.91 ± 2.21	
Birth weight [g]	3154 ± 424	2435 ± 612	< 0.0001
Gender			
Male	386 (58.0%)	106 (62.7%)	0.2609
Female	280 (42.0%)	63 (37.3%)	
Time of diagnosis			
Prenatal	512 (77.1%)	144 (85.2%)	0.0216
Postnatal	152 (22.9%)	25 (14.8%)	
Unknown	2	0	
Laterality of hernia			
Left	574 (86.1%)	130 (76.9%)	0.0026
Right	91 (13.7%)	39 (23.1%)	
Bilateral	1 (0.15%)	0 (0%)	
Size of defect			
A	63 (13.2%)	8 (7.8%)	0.1321
B	199 (41.8%)	36 (35.3%)	0.2243
C	176 (37.0%)	44 (43.1%)	0.2447
D	38 (8.0%)	14 (13.7%)	0.0659
Unknown	190	67	
ECMO treatment	231 (34.7%)	58 (34.3%)	0.9290
Mode of surgery			
Open surgery	511 (79.7%)	147 (91.3%)	0.0006
Minimal invasive surgery	130 (20.3%)	14 (8.7%)	
Unknown	25	8	
Repair of diaphragm			
Direct repair	181 (27.2%)	26 (15.4%)	0.0015
Patch repair	485 (72.8%)	143 (84.6%)	

Surviving patients without comorbidities only. Term (≥ 37 + 0 weeks of gestation) and preterm (< 37 + 0 weeks of gestation) children are compared, the level of significance is indicated. *P*-values below 0.05 are regarded as significant. Percentages apply to patients with known data only.

**Table 2:** Weight progression for term and preterm children without significant comorbidity

	Age at time of measurement	Samples size	***Z***-score mean	***Z***-score SD	***P***-value	Moderate underweight (***n***, %)	Severe underweight (***n***, %)	Moderate + severe underweight (***n***, %)	Overweight (***n***, %)
Term children	Birth	652	− 0.07	0.87	0.0561	10 (1.5%)	1 (0.2%)	11 (1.7%)	4 (0.6%)
	6 M	450	− 1.47	1.43	< 0.0001	80 (17.8%)	70 (15.6%)	150 (33.3%)	0
	12 M	436	− 1.16	1.29	< 0.0001	66 (15.1%)	40 (9.2%)	106 (24.3%)	1 (0.2%)
	2 Y	333	− 0.70	1.12	< 0.0001	37 (11.1%)	9 (2.7%)	46 (13.8%)	1 (0.3%)
	4 Y	253	− 0.86	1.13	< 0.0001	27 (10.7%)	10 (4,0%)	37 (14.6%)	2 (0.8%)
	6 Y	204	− 0.86	1.15	< 0.0001	24 (11.7%)	9 (4.4%)	33 (16.2%)	0
	10 Y	107	− 0.75	1.26	< 0.0001	14 (13.1%)	4 (3.7%)	18 (16.8%)	1 (0.9%)
Preterm children	Birth	164	− 0.02	0.93	0.7919	1 (0.6%)	0	1 (0.6%)	3 (1.8%)
	6 M	101	− 2.45	1.55	< 0.0001	25 (24.8%)	35 (34.7%)	60 (59.4%)	0
	12 M	101	− 1.72	1.38	< 0.0001	21 (20.8%)	17 (16.8%)	38 (37.6%)	0
	2 Y	71	− 1.41	1.39	< 0.0001	10 (14.1%)	9 (12.7%)	19 (26.8%)	0
	4 Y	63	− 1.10	1.07	< 0.0001	10 (15.9%)	1 (1.6%)	11 (17.5%)	0
	6 Y	55	− 1.48	1.31	< 0.0001	11 (20.0%)	6 (10.9%)	17 (30.9%)	0
	10 Y	39	− 1.21	1.12	< 0.0001	9 (23.1%)	2 (5.1%)	11 (28.2%)	0

Moderate underweight is defined as weight z-score between − 2 and − 3, whereas severe underweight is defined as weight z-score <  − 3. Overweight is defined as z-score > 2. Deviation of mean z-score from the normal population was tested for significance using the Z-test

**Table 3:** Length progression for term and preterm children without significant comorbidity

	Age at time of measurement	Sample size	*Z*-score mean	*Z*-score SD	*P*-value	Moderate stunting (*n*, %)	Severe stunting (*n*, %)	Moderate + severe stunting (*n*, %)	Tall stature (*n*, %)
Term children	Birth	600	+ 0.67	1.09	< 0.0001	3 (0.5%)	1 (0.2%)	4 (0.7%)	65 (10.8%)
	6 M	430	− 0.27	1.40	< 0.0001	38 (8.8%)	13 (3.0%)	51 (11.9%)	20 (4.7%)
	12 M	414	− 0.25	1.42	0.0003	32 (7.7%)	12 (2.9%)	44 (10.6%)	23 (5.6%)
	2 Y	320	− 0.46	1.10	< 0.0001	31 (9.7%)	0	31 (9.7%)	2 (0.6%)
	4 Y	248	− 0.53	1.01	< 0.0001	13 (5.2%)	4 (1.6%)	17 (6.9%)	1 (0.4%)
	6 Y	202	− 0.06	0.97	0.3592	6 (3,0%)	1 (0.5%)	7 (3.5%)	1 (0.5%)
	10 Y	105	− 0.12	1.03	0.2459	5 (4.8%)	0	5 (4.8%)	0
Preterm children	Birth	144	+ 0.48	1.08	< 0.0001	0	1 (0.7%)	1 (0.7%)	11 (7.6%)
	6 M	95	− 0.89	1.56	< 0.0001	16 (16.8%)	6 (6.3%)	22 (23.2%)	5 (5.3%)
	12 M	99	− 0.76	1.40	< 0.0001	13 (13.1%)	6 (6.1%)	19 (19.4%)	2 (2.0%)
	2 Y	70	− 0.89	1.50	< 0.0001	12 (17.1%)	5 (7.1%)	17 (24.3%)	3 (4.3%)
	4 Y	61	− 0.49	1.09	0.0004	4 (6.6%)	0	4 (6.6%)	1 (1.5%)
	6 Y	55	− 0.49	0.90	< 0.0001	4 (7.3%)	0	4 (7.3%)	0
	10 Y	38	− 0.48	0.84	0.0004	1 (2.6%)	0	1 (2.6%)	0

Moderate stunting is defined as height/length z-score between − 2 and − 3, whereas severe stunting is defined as height/length z-score <  − 3. Tall stature is defined as z-score > 2. Deviation of mean z-score from the normal population was tested for significance using the Z-test.

#### Underweight at 6 months of age improved within the first 2 years, stabilizing at a lower level thereafter

**Fig. 1:**
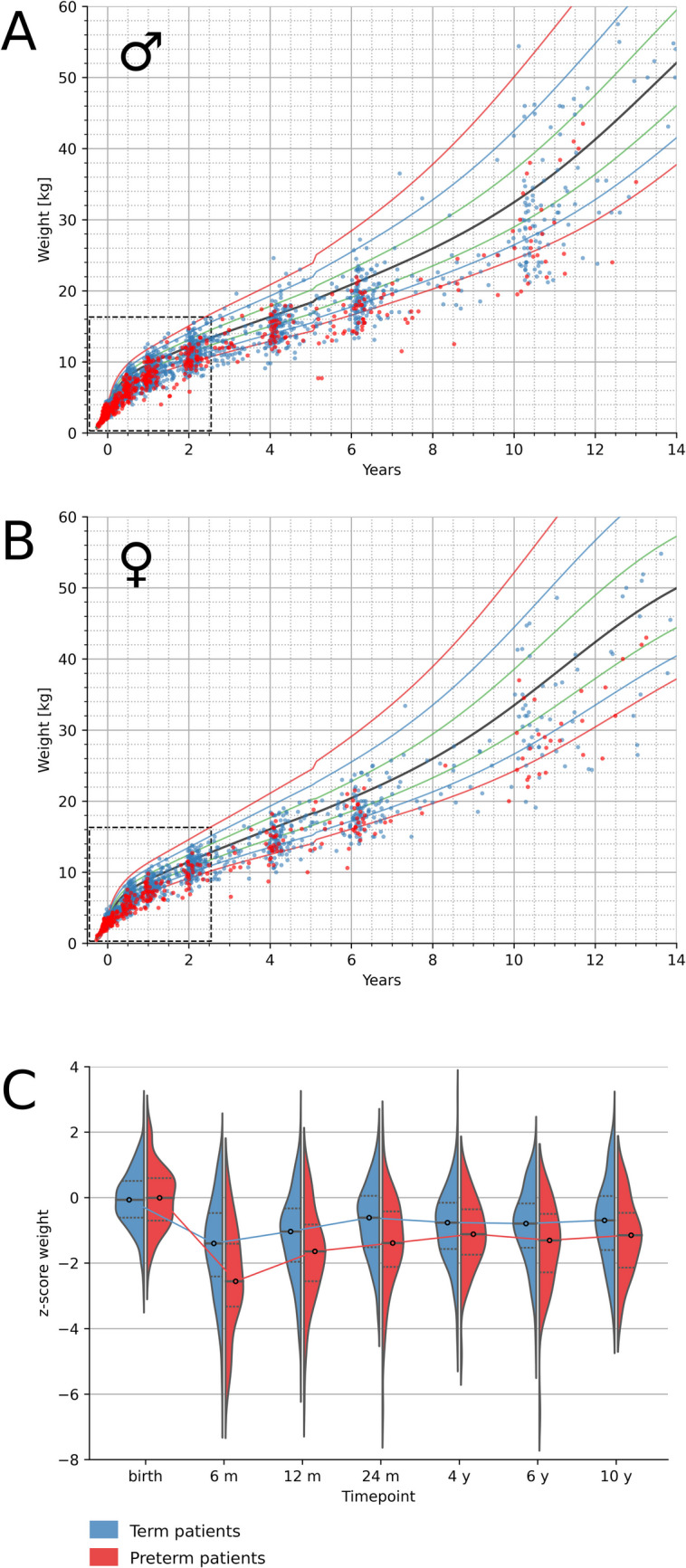
Weight progression for term and preterm children without significant comorbidity: **a** Weight for boys aged 0–14 years plotted on percentiles (3rd, 10th, 25th, 50th, 75th, 90th, and 97th). **b** Weight for girls aged 0–14 years plotted on percentiles (3rd, 10th, 25th, 50th, 75th, 90th, and 97th). Term infants are displayed as blue dots, preterm children (< 37 + 0 weeks of gestation) are displayed as red dots. For a magnification of the dashed area, see Online Resource 3. **c** Weight *z*-scores for term children (blue) and preterm children (red) displayed as violin plots. The dotted lines show the first quartile (Q1) and the third quartile (Q3) of the data, with a line at the median. The plots extend to show all data points. The width of the violin plot shows the frequency of occurrence

In surviving patients, the distribution of the measurements indicated a skew towards earlier time points, with increased frequency of data collection occurring in the initial months. In the older age groups, the number of available measurements decreased, therefore weight percentile curves only include children up to 14 years of age. Term children without significant comorbidities showed higher weights and faster catch-up than preterm children without significant comorbidities (Fig. 1, Table 2, and Online Resource 3). Longitudinal analysis of the data using an LMM also demonstrated a significant improvement in weight *z*-scores of term-born children between an early (6 months) and late (6 years) follow-up time point (Online Resources 4 and 5). During the first 6 months, however, there was a rapid, significant drop in the average *z*-score, which improved over the next 2 years. Weight remained continuously significantly lower than that of healthy children until the end of the evaluated measurement period for term and preterm children with CDH (Table 2). 

### Normalization of stature by 6 years of age in CDH patients

**Fig. 2:**
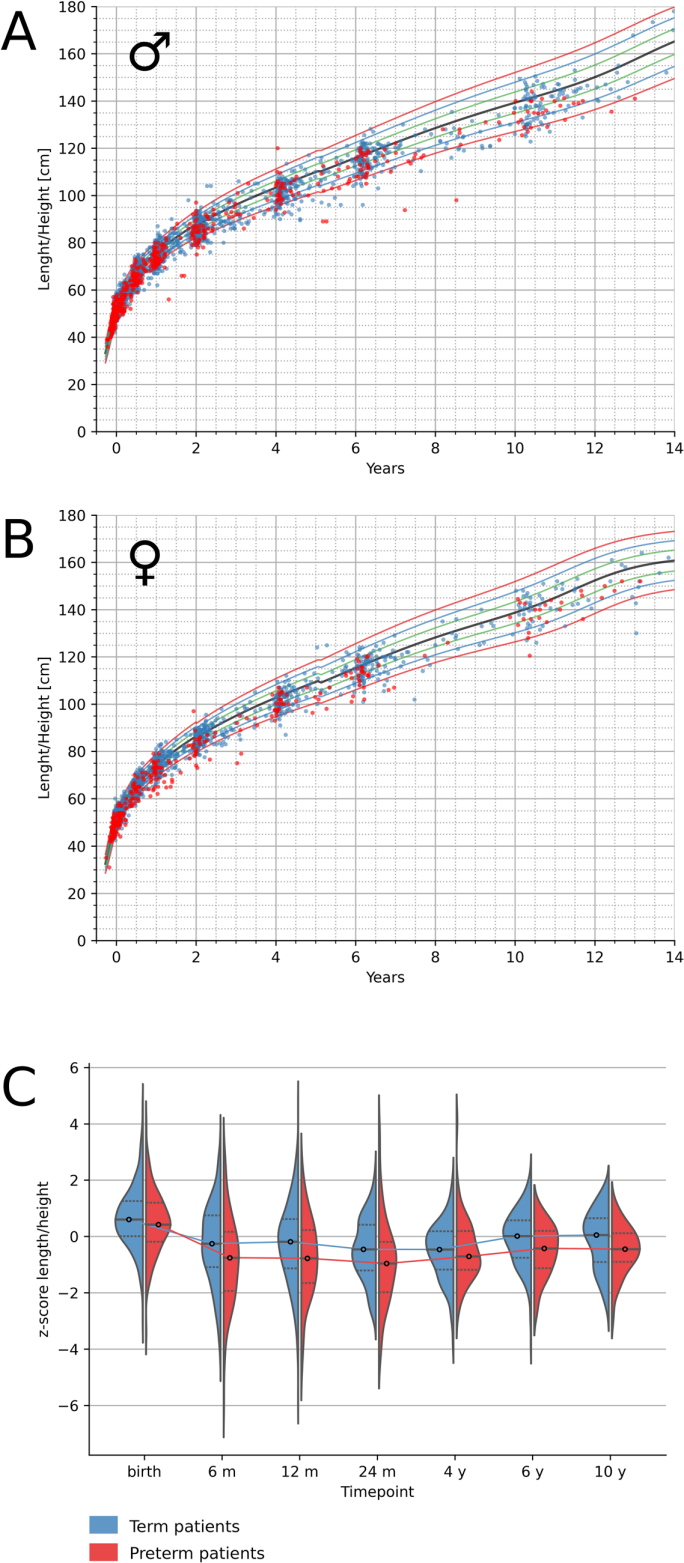
Length progression for term and preterm children without significant comorbidity:** a** Length for boys aged 0–14 years plotted on percentiles (3rd, 10th, 25th, 50th, 75th, 90th, and 97th). **b** Length for girls aged 0–14 years plotted on percentiles (3rd, 10th, 25th, 50th, 75th, 90th, and 97th). Term infants are displayed as blue dots, preterm children (< 37 + 0 weeks of gestation) are displayed as red dots. **c** Length *z*-scores for term children (blue) and preterm children (red) displayed as violin plots. The dotted lines show the first quartile (Q1) and the third quartile (Q3) of the data, with a line at the median. The plots extend to show all data points. The width of the violin plot shows the frequency of occurrence

Term children with CDH were significantly smaller than their healthy peers from the age of 6 months to 4 years. In older children, the difference was not statistically significant. Analyzing the group of term-born patients longitudinally showed a significant improvement of length *z*-score between the 6-month and 6-year time points (Online Resources 4 and 6). In contrast, preterm CDH children remained smaller than healthy children throughout the observed period (Fig. 2, Table 3). At birth, term CDH children seemed to be significantly larger than healthy children.

### Normalization of head circumference by 1 year of age

**Fig. 3:**
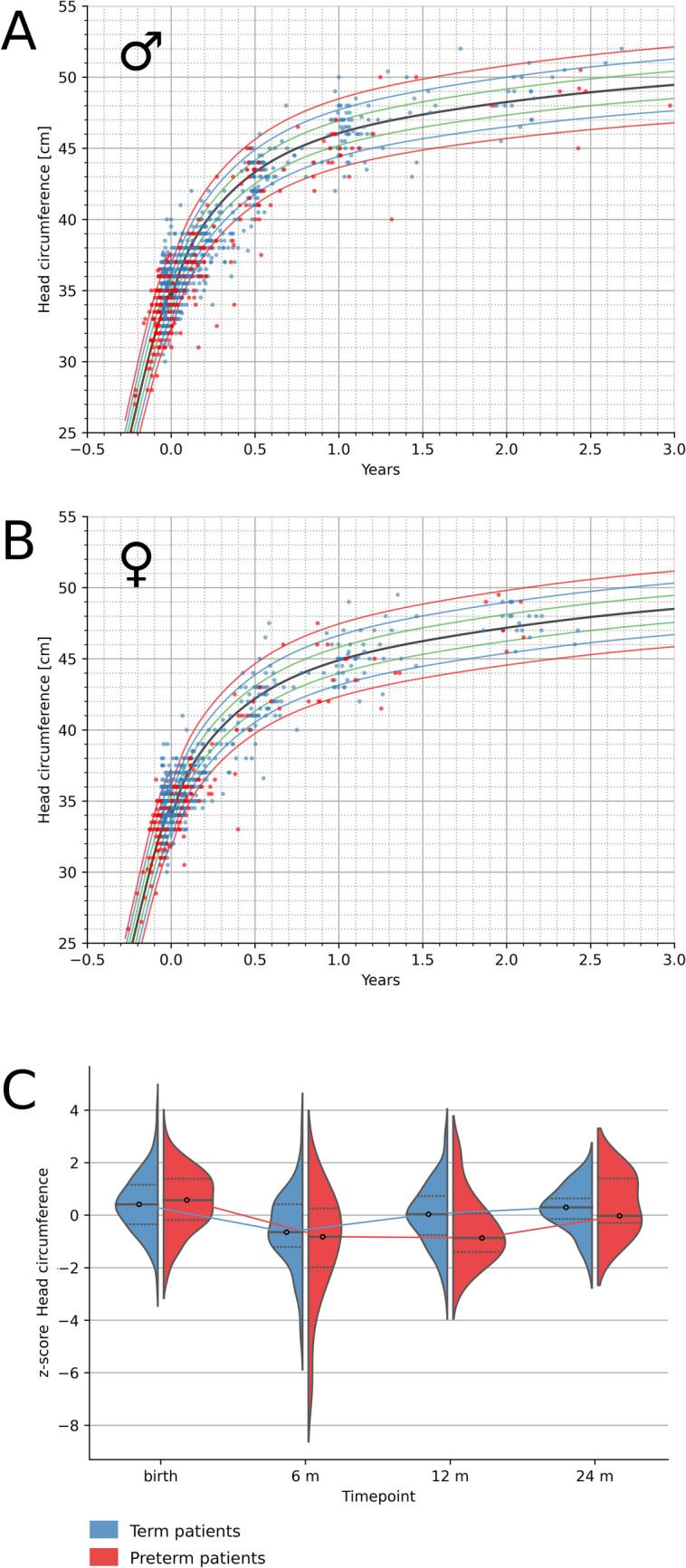
Head circumference progression for term and preterm children without significant comorbidity: **a** Head circumference for boys aged 0–3 years plotted on percentiles (3rd, 10th, 25th, 50th, 75th, 90th, and 97th). **b** HC for girls aged 0–3 years plotted on percentiles (3rd, 10th, 25th, 50th, 75th, 90th, and 97th). Term infants are displayed as blue dots, preterm children (< 37 + 0 weeks of gestation) are displayed as red dots. **c** HC *z*-scores for term children (blue) and preterm children (red) displayed as violin plots. The dotted lines show the first quartile (Q1) and the third quartile (Q3) of the data, with a line at the median. The plots extend to show all data points. The width of the violin plot shows the frequency of occurrence. HC = head circumference

HC tended to be significantly diminished at the early follow-up time point, catching up at 12 months in term CDH children and at 2 years in preterm CDH patients (Fig. 3 and OnlineResource 7).

### BMI remained low throughout childhood

Mean BMI was significantly lower both in term and in preterm children throughout the entire follow-up period in comparison to healthy children, with a minimum at the age of 6 months for both groups (OnlineResources 8 and 9).

### Major determinants of poor weight gain and growth were defect size and gestational age

Multiple linear regression analyses were used to assess the role of gender, gestational age, laterality of the defect, and defect size on weight and on length. For this purpose, growth data were used from the follow-up visits at 12 months ± 3 months and at 4 years ± 6 months.

As shown in OnlineResources 10 and 11, large defect size (C, D) was an independent predictor of poor weight and shorter length at the early and late time points, whereas gestational age was a predictor of poor growth at the early time point only.

### ECMO did not seem to influence weight and stature

In surviving children without relevant comorbidities, PSM was used to assess the potential impact of ECMO therapy on weight and height.

Comparison of the matched groups showed no significant effect of ECMO therapy on weight at 1 year (ECMO *z*-score: − 1.46 ± 1.12, non-ECMO *z*-score: − 1.072 ± 1.17, *p* = 0.0616), or at 4 years (ECMO *z*-score: − 1.02 ± 1.07, non-ECMO z-score: − 0.65 ± 1.14, *p* = 0.1382). Likewise, ECMO did not appear to be an independent variable associated with a reduced stature at 1 year of age (ECMO *z*-score: − 0.46 ± 1.56, non-ECMO *z*-score: − 0.21 ± 1.32, *p* = 0.3560), or at 4 years of age (ECMO *z*-score: − 0.72 ± 1.03, non-ECMO *z*-score: − 0.44 ± 1.02, *p* = 0.2305).

## Discussion

The present study analyzed 835 CDH patients without comorbidities out of 1247 treated at a single center from birth to 10 years of age. The main findings indicate a generally reduced *z*-score for weight, height, HC, and BMI during the first months of life. While *z*-scores of all growth measures improved over time, only height and HC recovered fully to the mean of the reference population. Despite initial improvements, in the early years, both weight and BMI remained substantially below those of the healthy reference cohort until the end of the study period. Diaphragmatic defect size and gestational age were demonstrated to be associated with growth restriction in these patients.

Several early aspects impede physiological enteral feeding patterns in CDH patients, like delayed start of oral and enteral nutrition. Restrictive volume management postoperatively leads to nutritional energy restriction, as seen in children with chronic heart disease after surgery [22]. At later ages, nutritional impediments can persist due to GERD, as well as acquired oral aversion. Moreover, many patients remain in a hypermetabolic state [13, 23]. Additionally, underlying pulmonary disease, including lung hypoplasia, compromises oxygenation and increases respiratory workload, thereby reducing the effective utilization of ingested calories [24, 25]. Particularly, in this cohort, large defect sizes were frequent, indicating a high prevalence of severe lung hypoplasia. Importantly, the exact influence of such pulmonary limitations on growth in CDH remains insufficiently characterized [24]. Altogether, these factors promote malnutrition in a significant proportion of CDH patients.

The present study revealed a maximum decline in weight *z*-score at the age of 6 months, with 33% of term and 59% of preterm patients being underweight, thereafter mean weight *z*-score improved. Underweight was still present in 17% of term-born and in 28% of preterm-born patients at the age of 10 years.

Leeuwen and colleagues analyzed growth of Australian children with CDH in the first year of life and found mean weight-for-age *z*-scores at a lower level (− 0.85 ± 1.32) at the age of 1 year [10]. Lower *z*-scores in the present cohort likely reflect the University Medical Center Mannheim’s role as a national ECMO referral center [26].

Furthermore, linear growth was impaired in the term CDH group, with the lowest *z*-score for height at 4 years of age. However, at 6 years and 10 years, the cohort no longer differed from the CDC reference collective. While the Dutch cohort also improved in growth after the age of 5 years, it remained smaller than their reference group of Dutch children until the end of the study at 12 years [13]. Notably, preterm CDH patients showed a greater and persistent height deficit than term peers through 10 years, but the prevalence of growth restriction declined over time, with no severe stunting thereafter.

In both groups, HC, a surrogate of brain growth [27], was smaller at the age of 6 months compared to the norm. Subsequently, catch-up growth led to normalization at 1 year and 2 years of age in term and preterm infants, respectively. A similar trend was found in the Australian cohort [10].

Regarding the BMI of CDH patients, a mean *z*-score of − 0.49 ± 1.08 was found in a Canadian study for patients aged 5.0–6.9 years [23], while our term cohort had a lower mean BMI *z*-score of − 1.37 ± 1.57 at 6 years of age. This difference is mainly due to a lower percentage of stunted patients in the present term cohort (3.5% vs. 14% in the Canadian cohort) at 6 years of age. Moreover, a slightly higher percentage of underweight children in the present cohort of term children (16.2% vs. 14% in the Canadian cohort) contributes to this effect.

The differences between our patients and the aforementioned cohorts in terms of weight, height, and BMI *z*-scores are possibly due to differences in terms of defect size and therefore disease severity or treatment algorithms.

Length and HC at birth appeared to be significantly larger than the reference cohort. This could be due to the use of the Fenton percentiles for term children < 40 weeks of gestation. Another reason could be the difficulty of reliably measuring critically ill children who require intensive care after birth. Of note, the selection of the respective growth reference influences outcomes, for example with regard to the thresholds for underweight and stunting. After the age of 6 months, the WHO weight percentiles are below the CDC percentiles. Conversely, the WHO length/height curves are mainly above the CDC’s [28].

A multiple linear regression analysis found gestational age and large defect size to be prognostic factors for poor weight gain at the age of 1 year, but only defect size remained relevant at 4 years. A similar correlation was obtained for reduced height at both time points. These findings are in line with Leeuwen et al., reporting a correlation for gestational age and height-for-age [13].

PSM showed no link between ECMO and growth, unlike studies not correcting for confounding factors [29]. ECMO likely reflects illness severity rather than being an independent risk factor, with our results possibly due to specialized care.

The CDH EURO Consortium consensus, a standardized protocol for CDH care, has improved survival rates but provides limited guidance on nutrition [5, 30]. An updated protocol addressing neonatal and long-term nutrition could reduce morbidity. Improved outcomes reported by Bairdain et al. for the Boston cohort, with weight *z*-scores improving from below − 2 (1990–2000) to − 0.4 at 12 months (2000–2010), highlight the potential of a consensus-based nutritional program [29, 31], and may also reduce costs, as shown for hospital malnutrition in other patient populations [32, 33].

A recent clinical consensus guideline for CDH nutrition recommends initiating parenteral nutrition within 24 h, with specific targets for calories, proteins, and lipids [34]. Enteral nutrition should begin post-repair, with individualized adjustments and thorough growth monitoring. Multidisciplinary follow-up after discharge is advised to optimize feeding. These guidelines mark a significant step toward standardizing nutritional support for CDH patients.

Publishing evidence-based guidelines for rare diseases is challenging, leading to conflicting recommendations. For example, while unrepaired hernia is often considered a contraindication for enteral nutrition [34], Larsen et al. found preoperative enteral feeding feasible and safe [35]. Similarly, early parenteral nutrition has shown benefits in CDH [36], however, the PEPaNIC trial suggests delayed initiation may improve outcomes in critically ill neonates [37, 38]. The relevance of these findings for CDH patients, at high risk of malnutrition, remains unclear. Future studies should assess whether the new guideline improves growth and neurocognitive outcomes, as seen for weight gain with standardized nutrition protocols in high-risk neonates after cardiac surgery [39]. As has been shown for the prediction of primary pulmonary hypertension and mortality in CDH patients, the use of artificial intelligence, such as machine learning, could also help to identify high-risk patients for malnutrition at an early stage [40].

### Strengths

Our study has several strengths. It comprises the largest single-center analysis of growth patterns of CDH patients to date, analyzing 835 out of 1247 consecutive patients and more than 9.000 measurements, thus providing stronger and more reliable results. The application of *z*-scores to evaluate the four major growth parameters, weight, length, HC, and BMI, facilitates the comparison with other cohorts and studies. WHO, CDC, and Fenton growth data were used as international reference standards for better comparability.

### Limitations

Our study has six main limitations. First, the single-center design may have influenced growth outcomes due to local medical and nutritional practices, furthermore, being a referral center for ECMO leads to a disproportionately high percentage of patients with large defects possibly influencing growth parameters, as explained in detail above. During the study period, a standardized protocol for intensive care and surgical management was introduced in November 2007, leading to modifications in treatment algorithms [30]. Second, as a longitudinal study, not all patients were followed until adulthood, and some have not yet reached adulthood or dropped out of the follow-up program very early. Third, measurement errors, particularly in small, restless children or in the early intensive care setting, cannot be excluded, and in rare cases, subgroup sizes were small enough to limit the robustness of distributional assumptions. Fourth, the use of CDC and WHO growth references was chosen to represent non-white, non-Central European children, providing inclusivity. However, this approach involved trade-offs, as these datasets include fewer Central European children than the current cohort, which may limit generalizability. Fifth, no cost analysis was performed, limiting inference on economic impact, but this is an interesting topic for future research.

Sixth, the application of Fenton preterm growth charts for all neonates born before gestational week 40+ 0 presents challenges in interpreting birth growth data. A high percentage of neonates are classified as significantly larger compared to their peers shortly after 40 weeks of gestation. Additionally, the use of the CDC, WHO, and Fenton calculations introduces apparent discontinuities in transitional zones of the different reference percentile curves at 0 and 5 years of age.

## Conclusion

The study analyzed growth patterns in a large cohort of CDH patients using international growth standards. The study showed that a relevant proportion of CDH patients experienced impaired growth in and beyond the first year of life. While height and HC normalized during childhood, weight and BMI remained significantly below average in a subgroup of patients up to the age of 10 years. The risk factor for poor weight gain and smaller stature at the age of 1 and 4 years was a greater defect size, while lower gestational age was only relevant at 1 year but not at 4 years of age. ECMO was not an independent risk factor. The results show that there is a significant risk of failure to thrive, yet there is also potential for improvement. A structured follow-up program that evaluates growth and optimizes nutrition when necessary is essential. Reducing malnutrition and its negative consequences, such as a higher risk of infection and a longer hospital stay, will most likely have a positive impact on both the individual patient and the healthcare system’s costs. Based on these findings, randomized controlled trials with a multicenter design should be conducted to reduce bias due to center-specific treatment algorithms and examine the effect of nutritional interventions. Future research could also explore genetic factors and long-term outcomes in adulthood to provide a comprehensive understanding of growth patterns in CDH patients. 

## Supplementary Information

Below is the link to the electronic supplementary material.

ESM 1 (PDF 33.9 KB)

ESM 2 (DOCX 24.1 KB)

ESM 3 (PNG 1.16 MB)

High Resolution Image (TIF 2.66 MB)

ESM 4 (DOCX 16.5 KB)

ESM 5 (PNG 0.98 MB)

ESM 6 (PNG 901 KB)

ESM 7 (DOCX 181 KB)

ESM 8 (PNG 1.46 MB)

ESM 8 (TIF 3.31 MB)

ESM 9 (DOCX 182 KB)

ESM 10 (DOCX 180 KB)

ESM 11 (DOCX 180 KB)

## Data Availability

The data that support the findings of this study are available from the corresponding author upon reasonable request. All data are anonymized.

## Abbreviations

BMI: Body mass index
CDC: Centers for Disease Control and Prevention
CDH: Congenital diaphragmatic hernia
ECMO: Extracorporeal membrane oxygenation
GERD: Gastroesophageal reflux disease
HC: Head circumference
LMS: Lambda, mu, sigma
PSM: Propensity score matching
REML: Restricted maximum likelihood
SD: Standard deviation
WHO: World Health Organization
