# Supplementary figures and images for: Growth challenges and recovery in 1247 children with congenital diaphragmatic hernia: a 10-year follow-up
Source: Eur J Pediatr. 2025 Nov 7;184(12):738. doi: 10.1007/s00431-025-06479-w (PMC12594663; doi:10.1007/s00431-025-06479-w)

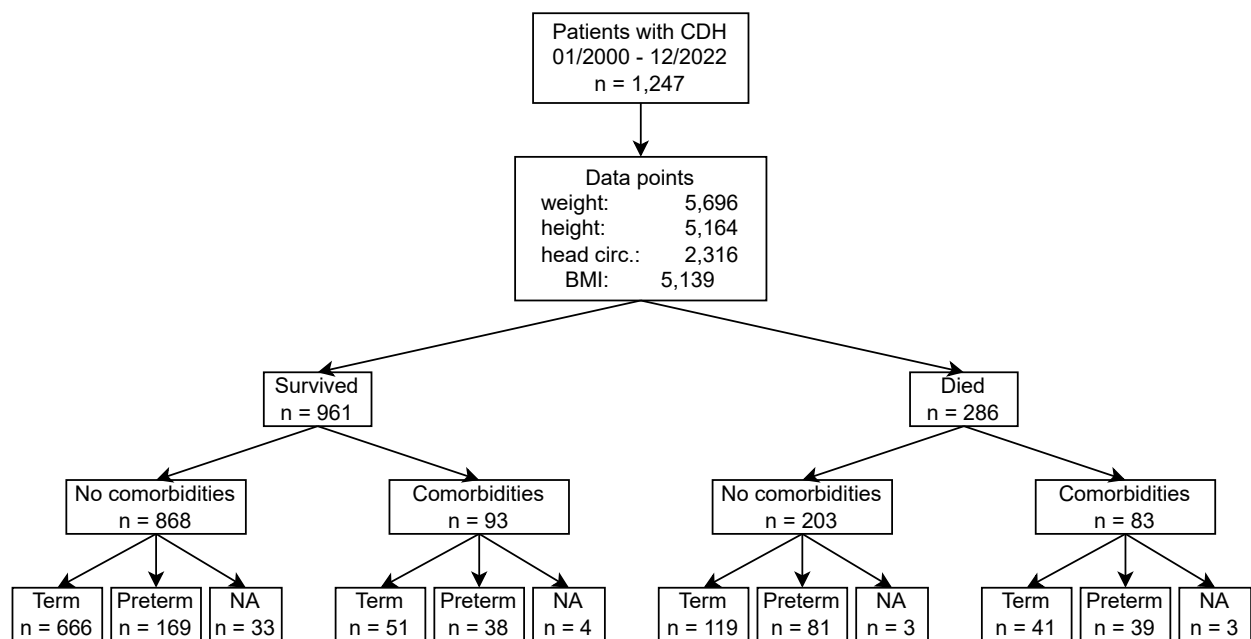

Supplement: Supplementary file 1 — (PDF 33.9 KB) [file 431_2025_6479_MOESM1_ESM.pdf]

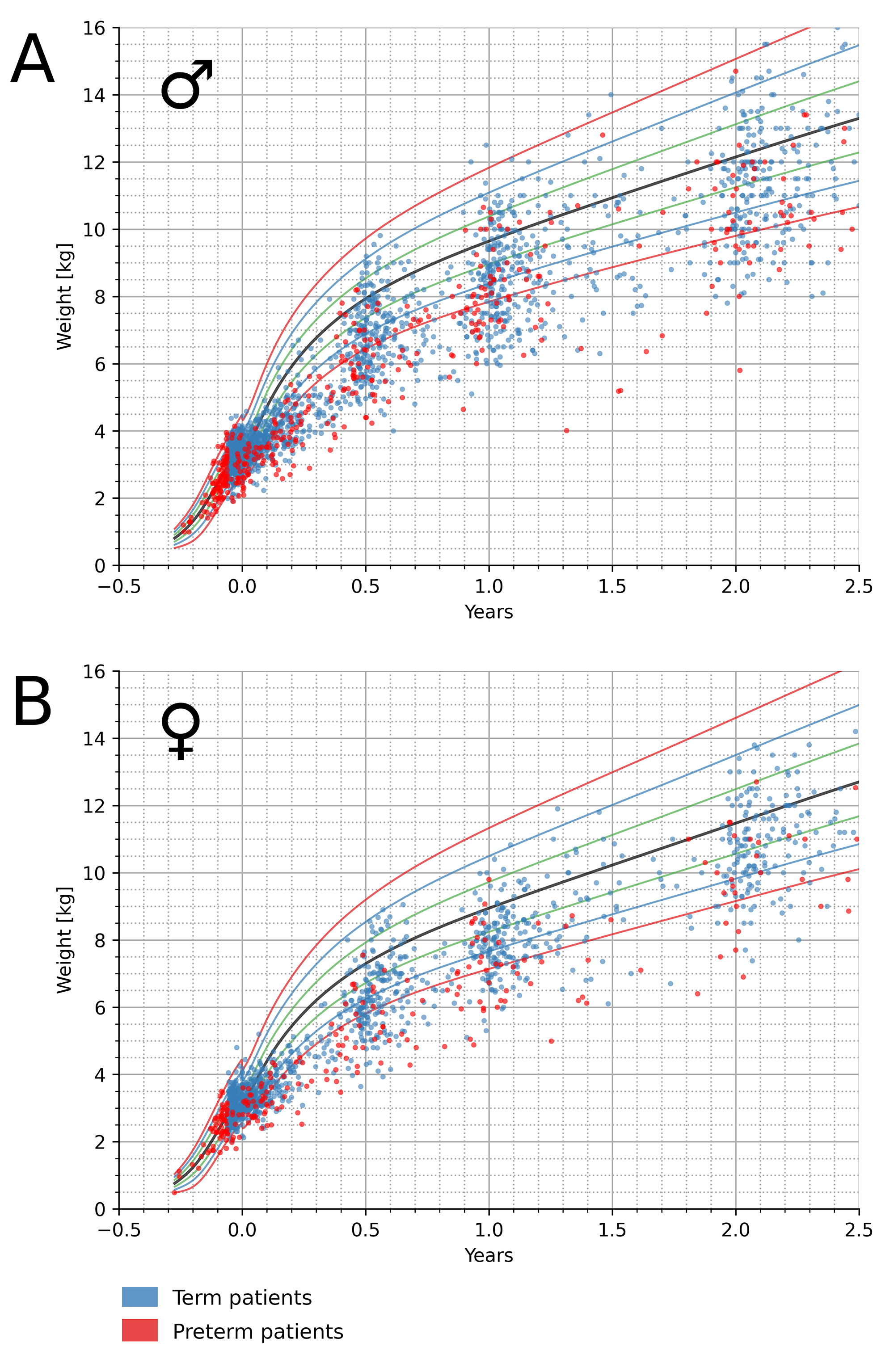

Supplement: Supplementary file 3 — (PNG 1.16 MB) [file 431_2025_6479_Fig4_ESM.png]

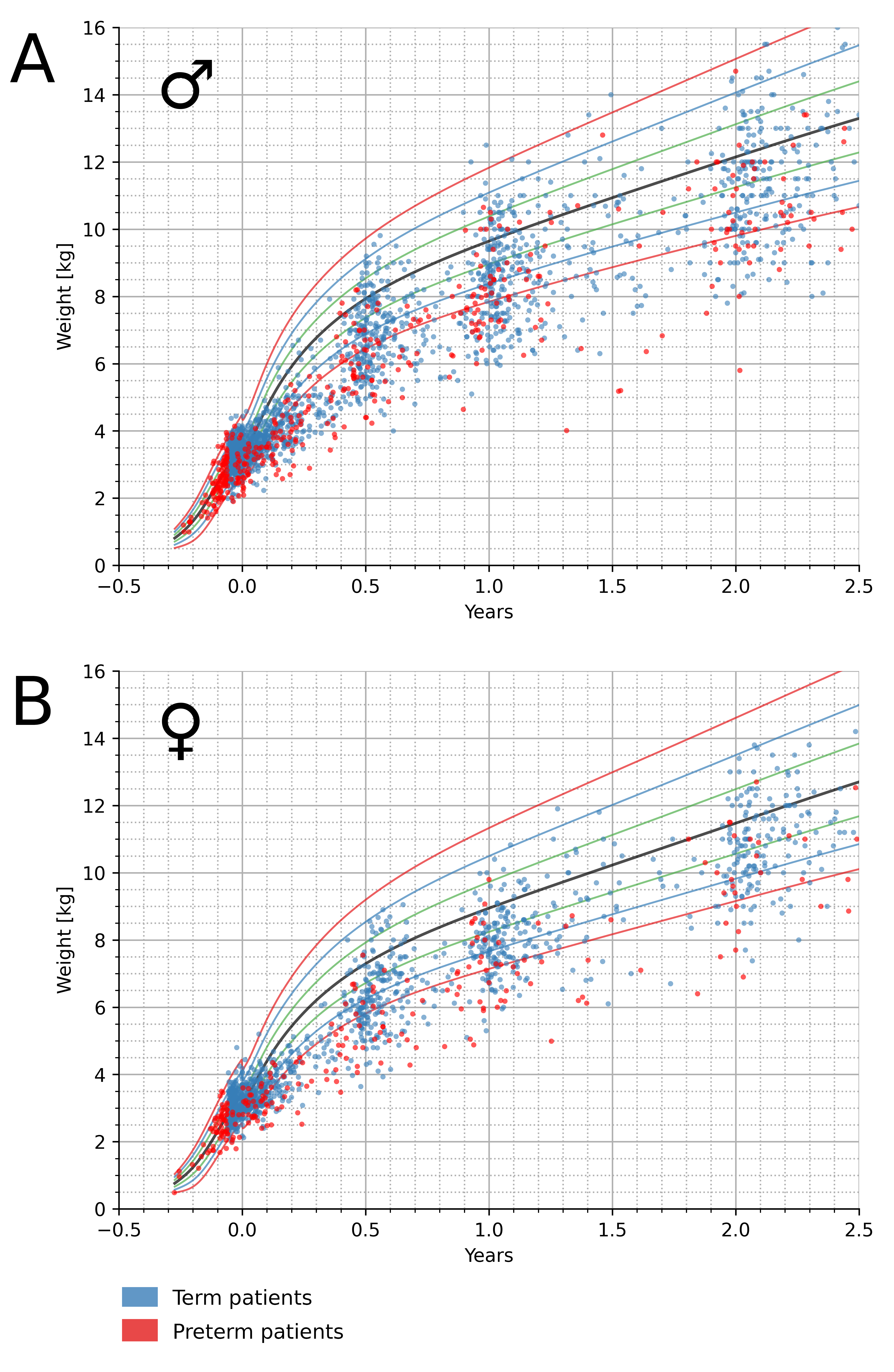

Supplement: Supplementary file 4 — (TIF 2.66 MB) [file 431_2025_6479_MOESM3_ESM.tif]

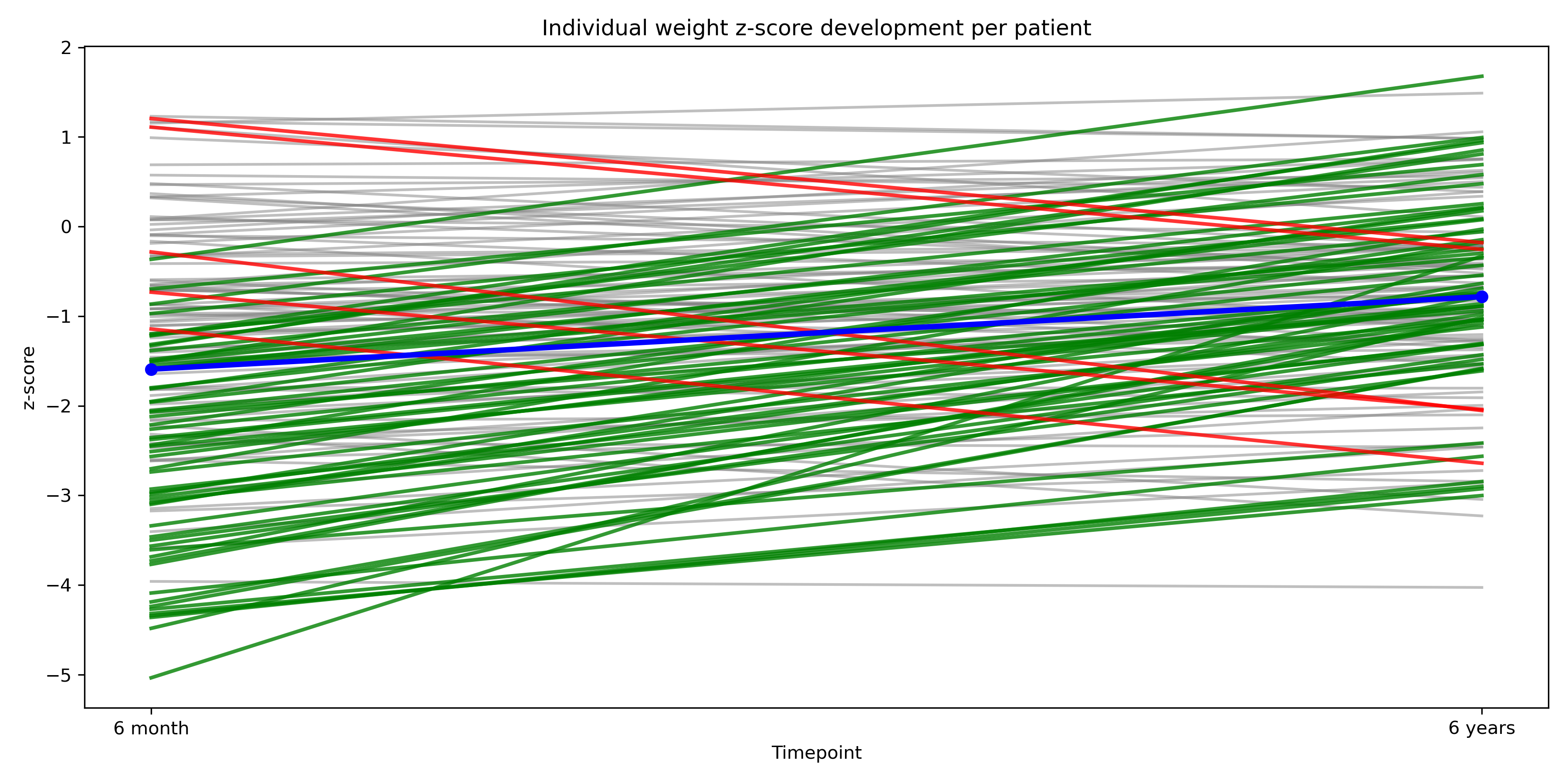

Supplement: Supplementary file 6 — (PNG 0.98 MB) [file 431_2025_6479_MOESM5_ESM.png]

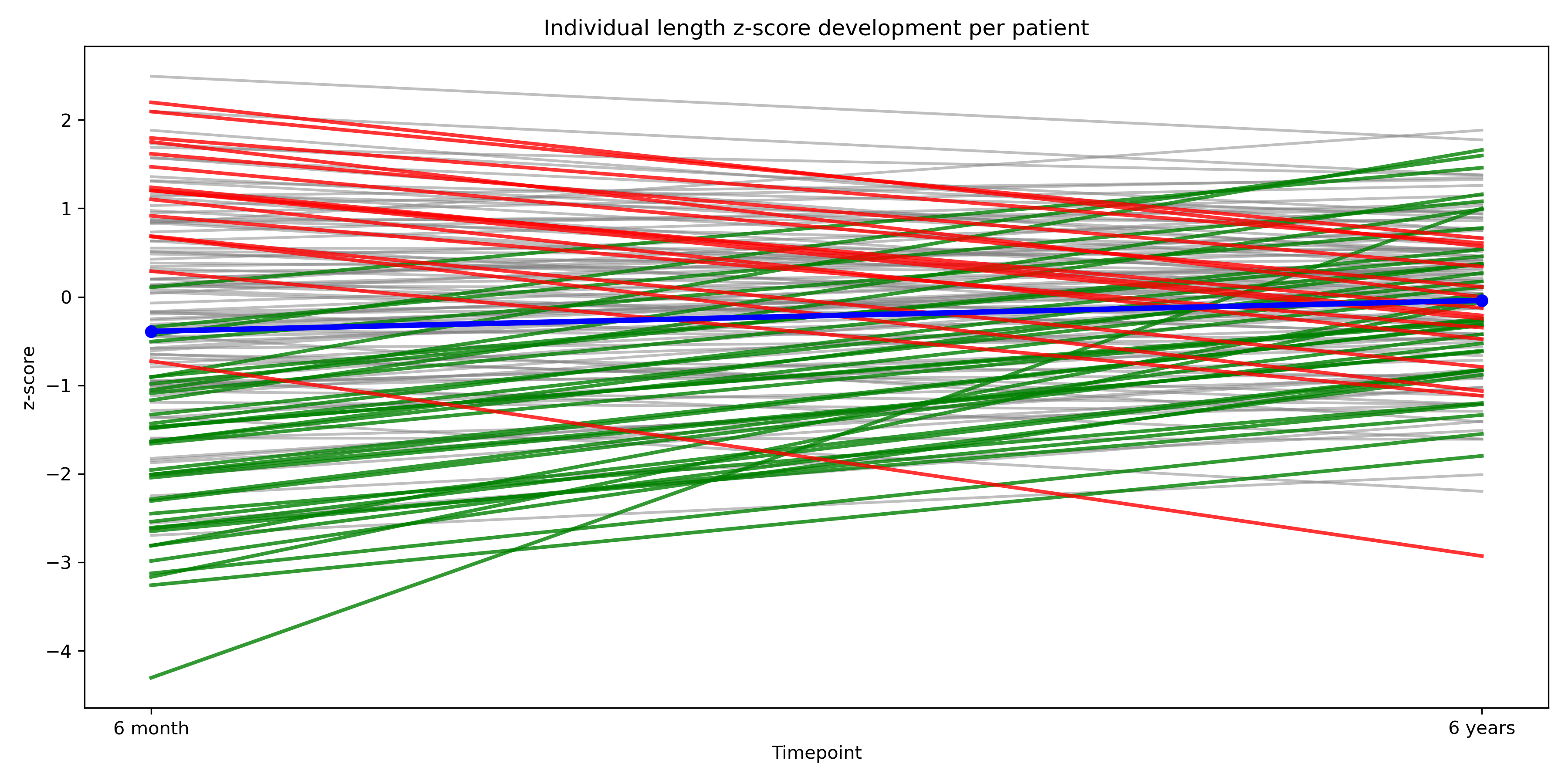

Supplement: Supplementary file 7 — (PNG 901 KB) [file 431_2025_6479_MOESM6_ESM.png]

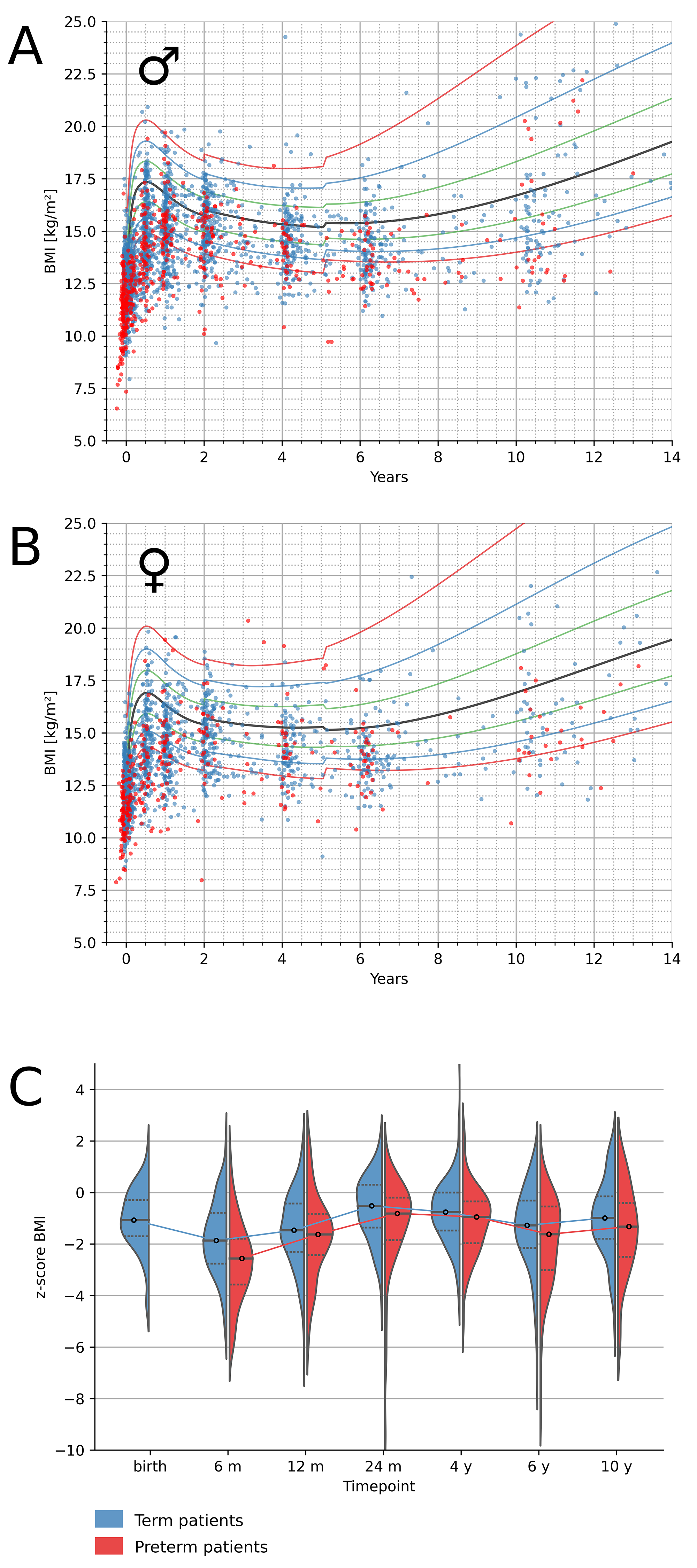

Supplement: Supplementary file 9 — (PNG 1.46 MB) [file 431_2025_6479_Fig5_ESM.png]

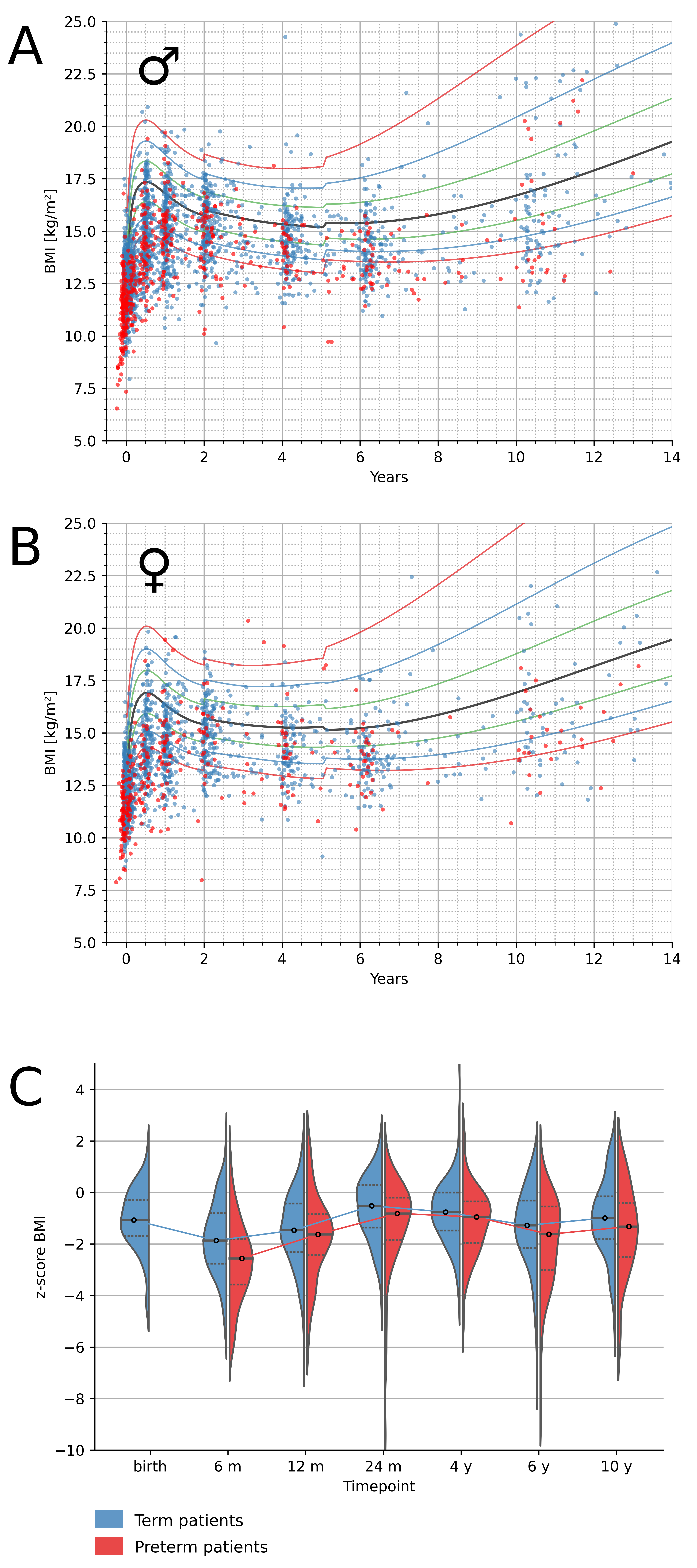

Supplement: Supplementary file 10 — (TIF 3.31 MB) [file 431_2025_6479_MOESM8_ESM.tif]
