# Supplementary material for: Growth challenges and recovery in 1247 children with congenital diaphragmatic hernia: a 10-year follow-up
Source: Eur J Pediatr. 2025 Nov 7;184(12):738. doi: 10.1007/s00431-025-06479-w (PMC12594663; doi:10.1007/s00431-025-06479-w)
Supplement: Supplementary file 2 — (DOCX 24.1 KB) [file 431_2025_6479_MOESM2_ESM.docx]

|  | No follow-up at 10 years | Completed follow-up at 10 years | p-value |
| --- | --- | --- | --- |
| total | 734 | 130 |  |
| gestational age | 37.83 ±2.06 | 37.62 ± 1.81 | 0.2861 |
| birth weight [g] | 3149 ± 425 | 2368 ± 608 |  |
| gender |  |  |  |
| *male* | 435 (59.3%) | 75 (57.7%) | 0.8110 |
| *female* | 299 (40.7%) | 55 (42.3%) |  |
| time of diagnosis |  |  |  |
| *prenatal* | 559 (76.8%) | 99 (78.0%) |  |
| *postnatal*  *unknown* | 169 (23.2%)  6 | 28 (22.0%)  3 | 0.8619 |
| size of defect |  |  |  |
| *A* | 74 (13.6%) | 6 (13.6%) | 0.8275 |
| *B* | 227 (41.7%) | 10 (22.7%) | **0.0213** |
| *C* | 195 (35.8%) | 25 (56.8%) | **0.0090** |
| *D*  *unknown* | 49 (9.0%)  189 | 3 (6.8%)  86 | 0.8318 |

**Online resource 2:** **Patient characteristics for follow up at 10 years**: Baseline characteristics of surviving patients without comorbidities, stratified by availability of follow-up data at 10 years of age. Patients with complete anthropometric data at 10 years were compared to those without follow-up at that timepoint. P-values below 0.05 are regarded as significant. Percentages apply to patients with known data, only.
