# Supplementary material for: Growth challenges and recovery in 1247 children with congenital diaphragmatic hernia: a 10-year follow-up
Source: Eur J Pediatr. 2025 Nov 7;184(12):738. doi: 10.1007/s00431-025-06479-w (PMC12594663; doi:10.1007/s00431-025-06479-w)
Supplement: Supplementary file 5 — (DOCX 16.5 KB) [file 431_2025_6479_MOESM4_ESM.docx]

A

           Mixed Linear Model Regression Results
=====================================================================

Model:            MixedLM    Dependent Variable:   zscore
No. Observations: 294        Method:               REML
No. Groups:       147        Scale:                0.5919
Min. group size:  2          Log-Likelihood:       -449.9254
Max. group size:  2          Converged:            Yes
Mean group size:  2.0

---------------------------------------------------------------------
                    Coef.  Std.Err. z    P>|z| [0.025 0.975]
---------------------------------------------------------------------

Intercept           -1.592  0.104 -15.351 0.000 -1.795 -1.389
time[T.Value_Jahre] 0.809    0.090    9.017 0.000  0.633  0.985
Group Var           0.989    0.276
=====================================================================

B

            Mixed Linear Model Regression Results
=====================================================================

Model:               MixedLM    Dependent Variable:   zscore
No. Observations:    284        Method:               REML
No. Groups:          142        Scale:                0.6782
Min. group size:     2          Log-Likelihood:       -427.1481
Max. group size:     2          Converged:            Yes
Mean group size:     2.0

---------------------------------------------------------------------
                     Coef. Std.Err.   z    P>|z| [0.025 0.975]
---------------------------------------------------------------------

Intercept           -0.389    0.097 -3.994 0.000 -0.580 -0.198
time[T.Value_Jahre]  0.349    0.098  3.569 0.000  0.157  0.540
Group Var            0.669    0.206
=====================================================================

**Online resource 4: Evolution of weight and length/height.** Linear mixed model for the evolution of weight, N=147 (a) and length/height, N=142 (b) between 6 months ± 2 months and 6 years ± 6 months of age for term born patients.

.
