# Supplementary material for: Growth challenges and recovery in 1247 children with congenital diaphragmatic hernia: a 10-year follow-up
Source: Eur J Pediatr. 2025 Nov 7;184(12):738. doi: 10.1007/s00431-025-06479-w (PMC12594663; doi:10.1007/s00431-025-06479-w)
Supplement: Supplementary file 8 — (DOCX 181 KB) [file 431_2025_6479_MOESM7_ESM.docx]

|  | **age at time of measurement** | **sample size** | **z-Score mean** | **z-Score SD** | **p-value** | **moderate microcephalus**  **(n, %)** | **severe microcephalus  (n, %)** | **moderate + severe microcephalus (n, %)** | **macrocephalus**  **(n, %)** |
| --- | --- | --- | --- | --- | --- | --- | --- | --- | --- |
| Term children | Birth | 557 | +0.43 | 1.10 | **< 0.0001** | 6 (1.1%) | 0 | **6 (1.1%)** | **39 (7.0%)** |
|  | 6 M | 152 | -0.55 | 1.38 | **< 0.0001** | 12 (7.9%) | 7 (4.6%) | **19 (12.5%)** | **4 (1.6%)** |
|  | 12 M | 103 | -0.03 | 1.15 | **0.8000** | 4 (3.9%) | 1 (1,0%) | **5 (4.9%)** | **3 (2.9%)** |
|  | 2 Y | 36 | 0.23 | 1.86 | **0.1034** | 0 | 0 | **0** | **0** |
|  | | | | | | | | | |
| Preterm children | Birth | 132 | +0.52 | 1.12 | **< 0.0001** | 3 (2.3%) | 0 | **3 (2.3%)** | **9 (6.8%)** |
|  | 6 M | 40 | -1.10 | 2.02 | **0.0006** | 4 (10.0%) | 6 (15.0%) | **10 (25.0%)** | **1 (2.5%)** |
|  | 12 M | 31 | -0.59 | 1.25 | **0.0093** | 2 (6.5%) | 0 | **2 (6.5%)** | **2 (6.5%)** |
|  | 2 Y | 7 | 0.40 | 1.12 | **0.3443** | 0 | 0 | **0%** | **0** |

**Online resource 7:** **Head circumference** **progression for term and preterm children without significant comorbidity**. Moderate microcephalus is defined as head circumference z-score between -2 and -3, whereas severe microcephalus is defined as head circumference z-score < -3. Macrocephalus is defined as z-score > 2. Deviation of mean z-score from the normal population was tested for significance using the Z-test.
