# Supplementary material for: Growth challenges and recovery in 1247 children with congenital diaphragmatic hernia: a 10-year follow-up
Source: Eur J Pediatr. 2025 Nov 7;184(12):738. doi: 10.1007/s00431-025-06479-w (PMC12594663; doi:10.1007/s00431-025-06479-w)
Supplement: Supplementary file 11 — (DOCX 182 KB) [file 431_2025_6479_MOESM9_ESM.docx]

|  | **age at time of measurement** | **sample size** | **z-score mean** | **z-score SD** | **p-value** | **moderate wasting**  **(n, %)** | **severe wasting**  **(n, %)** | **moderate + severe wasting (n, %)** | **overweight (n, %)** |
| --- | --- | --- | --- | --- | --- | --- | --- | --- | --- |
| Term children | Birth | 80 | -1.03 | 1.16 | **< 0.0001** | 5 (6.3%) | 4 (5.0%) | **9 (11.3%)** | **0** |
|  | 6 M | 426 | -1.78 | 1.42 | **< 0.0001** | 114 (26.8%) | 76 (17.8%) | **190 (44.6%)** | **2 (0.5%)** |
|  | 12 M | 414 | -1.42 | 1.43 | **< 0.0001** | 74 (17.9 %) | 55 (13.3 %) | **129 (31.2 %)** | **3 (0.7%)** |
|  | 2 Y | 320 | -0.61 | 1.25 | **< 0.0001** | 37 (11.6 %) | 9 (2.8 %) | **46 (14.4 %)** | **4 (1.3%)** |
|  | 4 Y | 248 | -0.76 | 1.21 | **< 0.0001** | 26 (10.5 %) | 10 (4.0 %) | **36 (14.5 %)** | **4 (1.6%)** |
|  | 6 Y | 202 | -1.37 | 1.57 | **< 0.0001** | 33 (16.3 %) | 26 (12.9 %) | **59 (29.2 %)** | **0** |
|  | 10 Y | 105 | -0.99 | 1.44 | **< 0.0001** | 11 (10.5 %) | 10 (9.5 %) | **21 (20.0 %)** | **0** |
|  | | | | | | | | | |
| Preterm children | Birth | - | - | - | **-** | - | - | **-** | **-** |
|  | 6 M | 95 | -2.69 | 1.54 | **< 0.0001** | 26 (27.4 %) | 41 (43.2 %) | **67 (70.5 %)** | **0** |
|  | 12 M | 95 | -1.69 | 1.58 | **< 0.0001** | 17 (17.9 %) | 19 (20.0 %) | **36 (37.9 %)** | **0** |
|  | 2 Y | 70 | -1.27 | 1.79 | **< 0.0001** | 9 (12.9 %) | 6 (8.6 %) | **15 (21.4 %)** | **0** |
|  | 4 Y | 61 | -1.12 | 1.30 | **< 0.0001** | 11 (18.0 %) | 4 (6.6 %) | **15 (24.6 %)** | **1 (1.6%)** |
|  | 6 Y | 55 | -1.96 | 1.74 | **< 0.0001** | 11 (20.0 %) | 14 (25.5 %) | **25 (45.5 %)** | **0** |
|  | 10 Y | 38 | -1.45 | 1.69 | **< 0.0001** | 8 (21.1 %) | 6 (15.8 %) | **14 (36.8 %)** | **0** |

**Online resource 9:** **BMI – term and preterm patients (≤ 36+6 weeks of gestation), without major comorbidities**. Moderate wasting is defined as BMI z-score between -2 and -3, whereas severe wasting is defined as BMI z-score < -3. Overweight is defined as z‑score > 2. Deviation of mean z-score from the normal population was tested for significance using the Z-test.
