# Supplementary material for: Growth challenges and recovery in 1247 children with congenital diaphragmatic hernia: a 10-year follow-up
Source: Eur J Pediatr. 2025 Nov 7;184(12):738. doi: 10.1007/s00431-025-06479-w (PMC12594663; doi:10.1007/s00431-025-06479-w)
Supplement: Supplementary file 12 — (DOCX 180 KB) [file 431_2025_6479_MOESM10_ESM.docx]

A

OLS Regression Results

==============================================================================

Dep. Variable: z-score weight 12 m R-squared: 0.154

Model: OLS Adj. R-squared: 0.141

Method: Least Squares F-statistic: 12.00

No. Observations: 403

==============================================================================

coef std err t P>|t| [0.025 0.975]

------------------------------------------------------------------------------

const -0.7837 0.095 -8.258 0.000 -0.970 -0.597

Sex 0.0327 0.120 0.272 0.786 -0.204 0.269

Right_Side 0.0031 0.171 0.018 0.985 -0.333 0.339

Size_A 0.2249 0.153 1.474 0.141 -0.075 0.525

Size_B 0.2665 0.094 2.823 0.005 0.081 0.452

Size_C -0.3977 0.098 -4.066 0.000 -0.590 -0.205

Size_D -0.8773 0.168 -5.237 0.000 -1.207 -0.548

Low_gest_age -0.1463 0.034 -4.302 0.000 -0.213 -0.079

==============================================================================

B

OLS Regression Results

==============================================================================

Dep. Variable: z-score weight 4 y R-squared: 0.088

Model: OLS Adj. R-squared: 0.064

Method: Least Squares F-statistic: 3.745

No. Observations: 240

==============================================================================

coef std err t P>|t| [0.025 0.975]

------------------------------------------------------------------------------

const -0.4543 0.112 -4.056 0.000 -0.675 -0.234

Sex -0.1970 0.133 -1.483 0.140 -0.459 0.065

Right_Side 0.0843 0.199 0.425 0.672 -0.307 0.475

Size_A 0.1841 0.175 1.054 0.293 -0.160 0.528

Size_B 0.1613 0.108 1.488 0.138 -0.052 0.375

Size_C -0.3566 0.109 -3.262 0.001 -0.572 -0.141

Size_D -0.4430 0.209 -2.122 0.035 -0.854 -0.032

Low_gest_age -0.0646 0.037 -1.742 0.083 -0.138 0.008

==============================================================================

**Online resource 10: Predictors of low weight at 1 and 4 years**. Linear regression model of predictors of low weight at 12 months ± 3 months, N = 403 (a) and at 4 years ± 6 months of age, N = 240 (b). Lower gestational age (Low_gest_age) is indicated as the difference to 40 weeks.
