# Supplementary material for: Growth challenges and recovery in 1247 children with congenital diaphragmatic hernia: a 10-year follow-up
Source: Eur J Pediatr. 2025 Nov 7;184(12):738. doi: 10.1007/s00431-025-06479-w (PMC12594663; doi:10.1007/s00431-025-06479-w)
Supplement: Supplementary file 13 — (DOCX 180 KB) [file 431_2025_6479_MOESM11_ESM.docx]

A

OLS Regression Results

==============================================================================

Dep. Variable: z-score length 12 m R-squared: 0.096

Model: OLS Adj. R-squared: 0.081

Method: Least Squares F-statistic: 6.723

No. Observations: 389

==============================================================================

coef std err t P>|t| [0.025 0.975]

------------------------------------------------------------------------------

const -0.1534 0.106 -1.449 0.148 -0.361 0.055

Sex 0.0779 0.134 0.582 0.561 -0.185 0.341

Right_Side -0.1055 0.192 -0.548 0.584 -0.484 0.273

Size_A 0.0531 0.169 0.313 0.754 -0.280 0.386

Size_B 0.4343 0.105 4.129 0.000 0.227 0.641

Size_C 0.0428 0.109 0.394 0.694 -0.171 0.257

Size_D -0.6836 0.189 -3.622 0.000 -1.055 -0.312

Low_gest_age -0.1310 0.038 -3.460 0.001 -0.205 -0.057

==============================================================================

B

OLS Regression Results

==============================================================================

Dep. Variable: Value R-squared: 0.042

Model: OLS Adj. R-squared: 0.017

Method: Least Squares F-statistic: 1.661

No. Observations: 236

==============================================================================

coef std err t P>|t| [0.025 0.975]

------------------------------------------------------------------------------

const -0.3763 0.103 -3.646 0.000 -0.580 -0.173

Sex 0.0642 0.123 0.521 0.603 -0.179 0.307

Right_Side 0.3201 0.185 1.734 0.084 -0.044 0.684

Size_A -0.2130 0.160 -1.327 0.186 -0.529 0.103

Size_B 0.0824 0.100 0.824 0.411 -0.115 0.279

Size_C -0.2545 0.101 -2.523 0.012 -0.453 -0.056

Size_D 0.0088 0.192 0.046 0.963 -0.369 0.387

Low_gest_age -0.0178 0.034 -0.517 0.606 -0.086 0.050

==============================================================================

**Online resource 11: Predictors of small stature at 1 and 4 years.** Linear regression model of predictors of small stature at 12 months ± 3 months, N = 389 (a) and at 4 years ± 6 months of age, N = 236 (b). Lower gestational age (Low_gest_age) is indicated as the difference to 40 weeks.
